# Supplementary material for: Non-neutralizing antibodies targeting the immunogenic regions of HIV-1 envelope reduce mucosal infection and virus burden in humanized mice
Source: PLoS Pathog. 2022 Jan 5;18(1):e1010183. doi: 10.1371/journal.ppat.1010183 (PMC8765624; doi:10.1371/journal.ppat.1010183)
Supplement: S1 Fig — Each mice was given V2i mAb 2158 and V3 mAb 2219 (700 μg per mAb) intraperitoneally. Plasma was collected and monitored for the concentration of V2i mAb 2158 and V3 mAb 2219 from day 0 to day 14 using ELISA with V1V2-1FD6 or peptide V3 antigens and the respective mAbs as standard. Based on the average values, the half life was estimated to be >14 days for V2i mAb 2158 and 11 days for V3 mAb 2219. (PDF) [file ppat.1010183.s001.pdf]

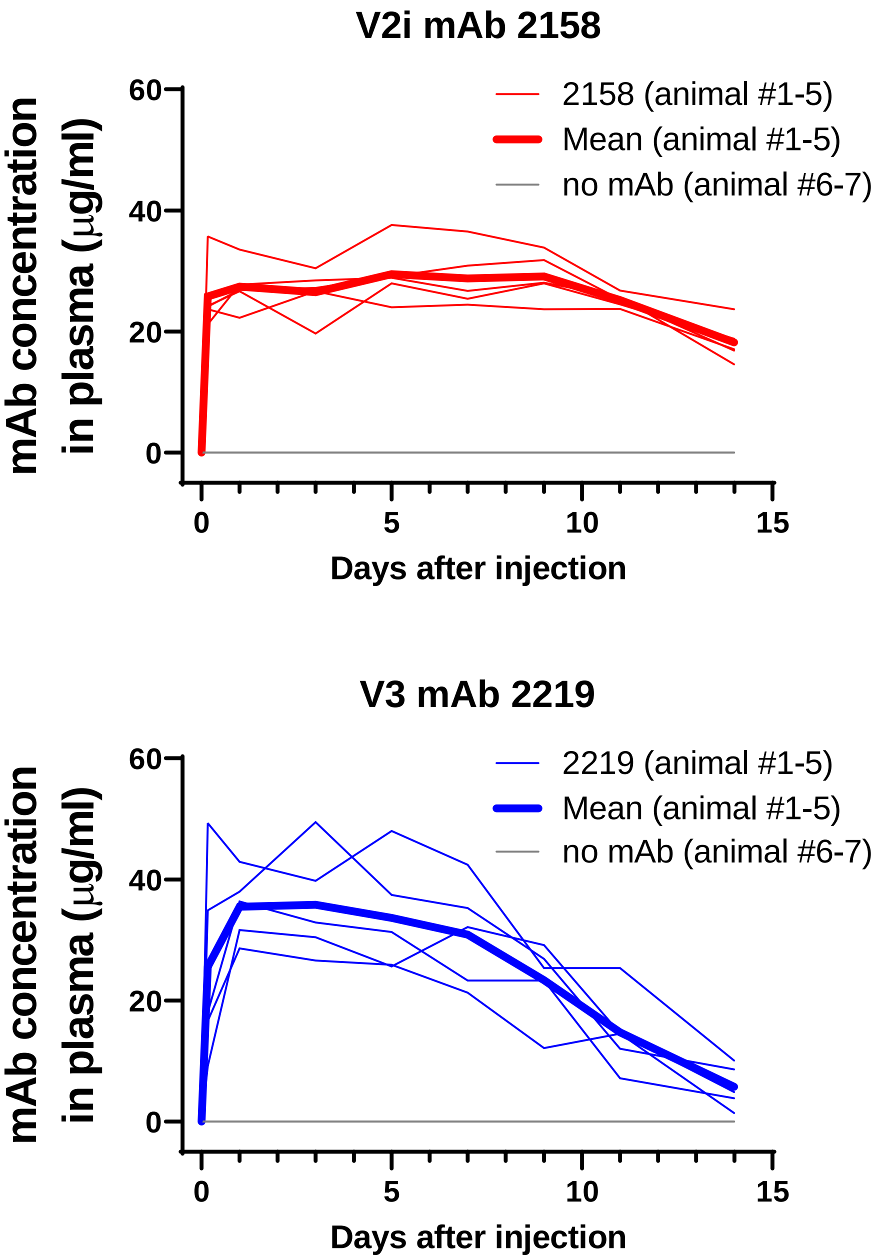

Fig S1: Pharmacokinetic study of human V2i mAb 2158 and V3 mAb 2219 in NSG mice.

Each mice was given V2i mAb 2158 and V3 mAb 2219 (700 µg per mAb) intraperitoneally. Plasma was collected and monitored for the concentration of V2i mAb 2158 and V3 mAb 2219 from day 0 to day 14 using ELISA with V1V2-1FD6 or peptide V3 antigens and the respective mAbs as standard. Based on the average values, the half life was estimated to be >14 days for V2i mAb 2158 and 11 days for V3 mAb 2219.
