# Supplementary material for: Non-neutralizing antibodies targeting the immunogenic regions of HIV-1 envelope reduce mucosal infection and virus burden in humanized mice
Source: PLoS Pathog. 2022 Jan 5;18(1):e1010183. doi: 10.1371/journal.ppat.1010183 (PMC8765624; doi:10.1371/journal.ppat.1010183)
Supplement: S2 Fig — A) Plasma vRNA loads from day 0 to day 9 in mice that received control mAb 860, V2i mAb 2158, or V3 mAb 2219. Individual mouse data from Fig 1B are shown. B) Relative levels of cell-associated vRNA in bone marrow and mesenteric lymph nodes collected at day 9 from animals in panel A. C) Relative vDNA levels in bone marrow and mesenteric lymph nodes from animals in panel A. *, p <0.05 by Kruskal-Wallis one-way ANOVA test with Dunn’s multiple comparison. Significant differences are marked, the other comparisons show no significant difference. (PDF) [file ppat.1010183.s002.pdf]

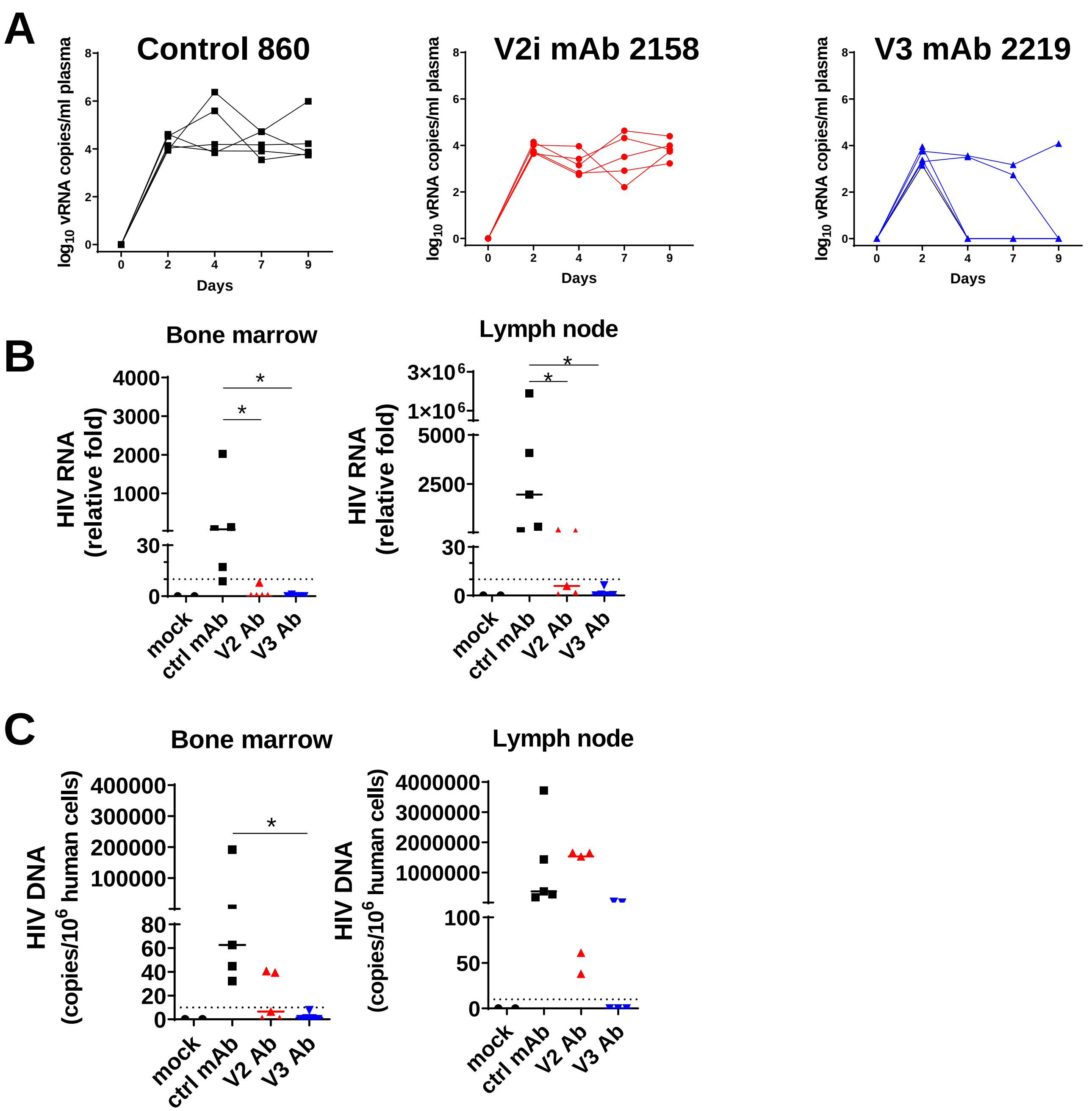

Fig S2: Reduced levels of plasma vRNA and cell-associated vRNA and vDNA in CD34+ engrafted humanized mice treated with V2i or V3 mAbs and infected with JRFL IMC.

A) Plasma vRNA loads from day 0 to day 9 in mice that received control mAb 860, V2i mAb 2158, or V3 mAb 2219. Individual mouse data from Figure 1B are shown.

B) Relative levels of cell-associated vRNA in bone marrow and mesenteric lymph nodes collected at day 9 from animals in panel A.

C) Relative vDNA levels in bone marrow and mesenteric lymph nodes from animals in panel A.

*, $p < 0.05$ by Kruskal-Wallis one-way ANOVA test with Dunn's multiple comparison. Significant differences are marked, the other comparisons show no significant difference.
