# Supplementary material for: Non-neutralizing antibodies targeting the immunogenic regions of HIV-1 envelope reduce mucosal infection and virus burden in humanized mice
Source: PLoS Pathog. 2022 Jan 5;18(1):e1010183. doi: 10.1371/journal.ppat.1010183 (PMC8765624; doi:10.1371/journal.ppat.1010183)
Supplement: S3 Fig — A) FcγRIIIA signaling was measured by co-incubating JRFL Δvpu-nucleofected Jurkat cells with Jur-γRIIIa luciferase reporter cells in the presence of V2i, V3, or control mAbs. CD4-binding site mAb b12 served as a positive control. RLU: relative light unit. B) ADCC activity was determined using CD4+ CEM.NKr target cells that were coated with recombinant gp120 JRFL, treated with V2i, V3, or control mAbs, and incubated with PBMCs as effector cells. V2i mAb, cradle-type V3 mAbs 2219 and 2557, and ladle-type V3 mAb 391/95 were tested along with anti-C1C2 mAb A32-3As (positive control) and purified human IgG (negative control). C) ADCC activity was examined in a second assay in which NKR24 luciferase reporter cells were infected with virus for 3 to 4 days, combined with the effector cells, human CD16+ NK cell line KHYG-1, at an effector-to-target cell ratio of 5:1, and incubated with serially diluted mAbs for 8 hours. The NKR24 target cell viability was measured by luciferase activity. PGT121 was used as a positive control. D) ADCC activity was also determined against primary CD4+ T cells infected with JRFL IMC or the Nef and Vpu-deleted counterpart. Binding of 2158 and 2219 to virus- vs mock-infected target cells was first examined by flow cytometry (top). Other V2i and V3 mAbs were tested for comparison. 3BNC117 (anti-CD4bs) and A32 (anti-C1C2) served as positive controls. ADCC were subsequently measured with PBMC effector cells at an effector-to-target ratio of 10:1 (bottom). Viability dye was used to measure cytotoxicity against infected target cells. Each mAb was tested at 5 μg/mL. E) Summary of experimental parameters and conditions for FcγRIIIA signaling and ADCC assays in Panels A-D. (PDF) [file ppat.1010183.s003.pdf]

Fig S3

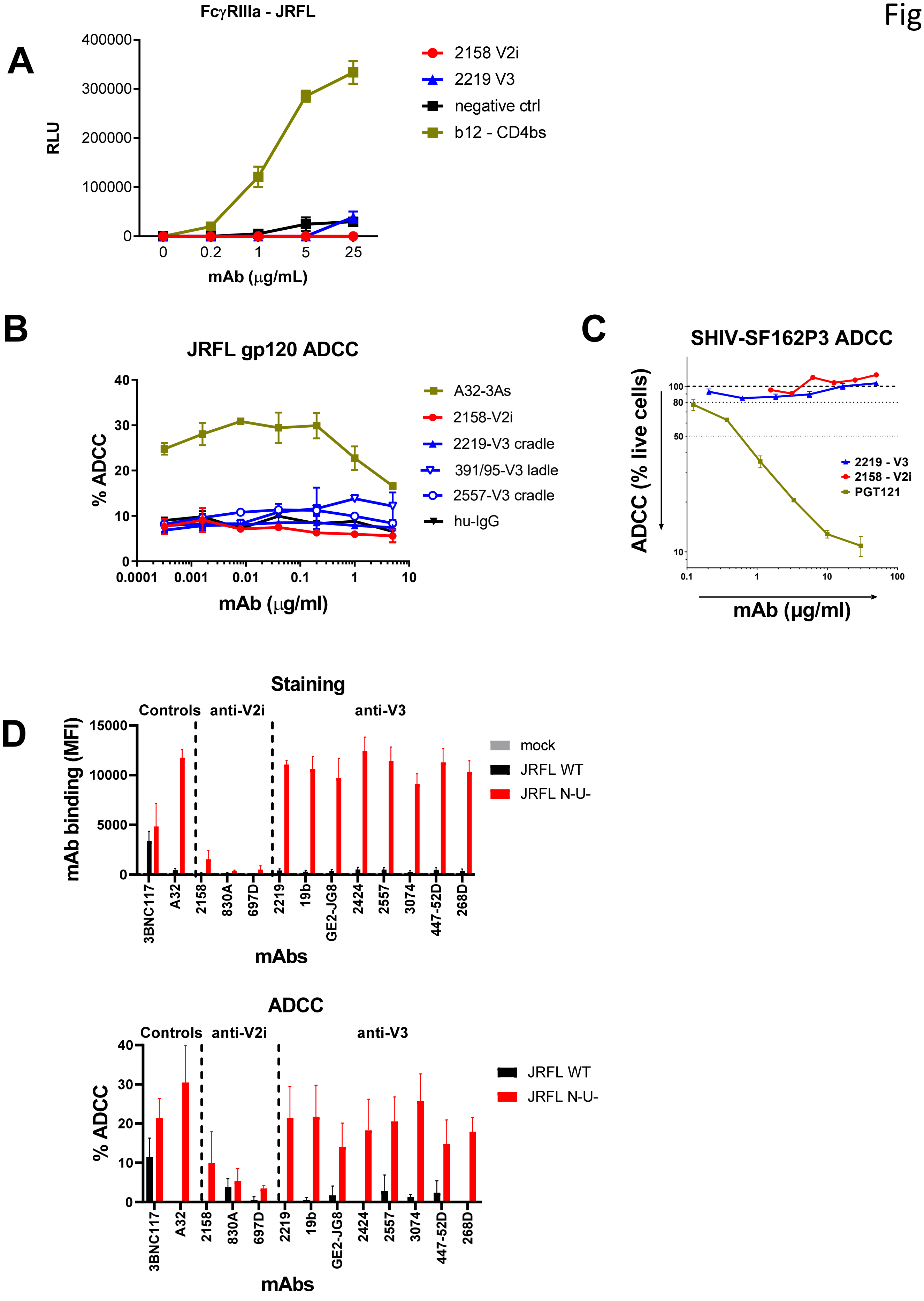
A
FcγRIIIa - JRFL
RLU
400000
300000
200000
100000
0
0
0.2
1
5
25
mAb (μg/mL)
2158 V2i
2219 V3
negative ctrl
b12 - CD4bs
B
JRFL gp120 ADCC
% ADCC
40
30
20
10
0
0.0001
0.001
0.01
0.1
1
10
mAb (μg/ml)
A32-3As
2158-V2i
2219-V3 cradle
391/95-V3 ladle
2557-V3 cradle
hu-IgG
C
SHIV-SF162P3 ADCC
ADCC (% live cells)
100
80
50
10
0.1
1
10
100
mAb (μg/ml)
2219 - V3
2158 - V2i
PGT121
D
Staining
Controls
anti-V2i
anti-V3
mAb binding (MFI)
15000
10000
5000
0
3BNC117
A32
2158
830A
697D
2219
19b
GE2-JG8
2424
2557
3074
447-52D
268D
mAbs
mock
JRFL WT
JRFL N-U-
ADCC
Controls
anti-V2i
anti-V3
% ADCC
40
30
20
10
0
3BNC117
A32
2158
830A
697D
2219
19b
GE2-JG8
2424
2557
3074
447-52D
268D
mAbs
JRFL WT
JRFL N-U-

E

Assay	Target cell	Virus or Env	Effector cells	E/T ratio	Readout
FcγRIIIA signaling (panel A)	Nucleofected Tetherin^{high} Jurkat cells	HIV-1 pNL4.3 Δvpu construct with JRFL Env and mCherry reporter	FcγRIIIA^{+} Jurkat-derived cells (Jur-γRIIIA) with firefly luciferase reporter under NFAT-responsive promoter	2:1	Luciferase activity in the effector cells induced by FcγRIIIA engagement; Promega luciferase kit
Fluorescent dye uptake ADCC assay (panel B)	gp120-coated CD4^{+} CEM.NKr cells labeled with CSFE and Fixable Viability Dye eFluor660	Recombinant monomeric JRFL gp120 protein	Pooled primary PBMCs from 8 HIV-negative donors	30:1	Fluorescent dye uptake by apoptotic target cells ; Flow cytometry to measure % eFluor660^{+} target cells
Luciferase ADCC assay (panel C)	Virus-infected NKR24 luciferase reporter cells	SHIV-SF162 P3	Human CD16^{+} NK cell line KHYG-1	5:1	Luciferase activity as indicator of target cell viability; Promega luciferase kit
Infected cell elimination (ICE) ADCC assay (panel D)	Virus-infected primary CD4^{+} T cells	JRFL infectious molecular clone or its Nef and Vpu-defective clone	PBMC from healthy HIV-negative individuals	10:1	Decrease of the infected (p24^{+}) cell population as detected by flow cytometry

E/T ratio: effector to target ratio

Fig S3: V2i mAb 2158 and V3 mAb 2219 lack the ability to mediate FcγRIIIa signaling and ADCC activity.

A) FcγRIIIA signaling was measured by co-incubating JRFL Δvpu-nucleofected Jurkat cells with Jur-γRIIIa luciferase reporter cells in the presence of V2i, V3, or control mAbs. CD4-binding site mAb b12 served as a positive control. RLU: relative light unit.

B) ADCC activity was determined using $CD4^+$ CEM.NKr target cells that were coated with recombinant gp120 JRFL, treated with V2i, V3, or control mAbs, and incubated with PBMCs as effector cells. V2i mAb, cradle-type V3 mAbs 2219 and 2557, and ladle-type V3 mAb 391/95 were tested along with anti-C1C2 mAb A32-3As (positive control) and purified human IgG (negative control).

C) ADCC activity was examined in a second assay in which NKR24 luciferase reporter cells were infected with virus for 3 to 4 days, combined with the effector cells, human $CD16^+$ NK cell line KHYG-1, at an effector-to-target cell ratio of 5:1, and incubated with serially diluted mAbs for 8 hours. The NKR24 target cell viability was measured by luciferase activity. PGT121 was used as a positive control.

D) ADCC activity was also determined against primary CD4+ T cells infected with JRFL IMC or the Nef and Vpu-deleted counterpart. Binding of 2158 and 2219 to virus- vs mock-infected target cells was first examined by flow cytometry (top). Other V2i and V3 mAbs were tested for comparison. 3BNC117 (anti-CD4bs) and A32 (anti-C1C2) served as positive controls. ADCC were subsequently measured with PBMC effector cells at an effector-to-target ratio of 10:1 (bottom). Viability dye was used to measure cytotoxicity against infected target cells. Each mAb was tested at 5 μg/mL.

E) Summary of experimental parameters and conditions for FcγRIIIA signaling and ADCC assays in Panels A-D.
