# Supplementary material for: Non-neutralizing antibodies targeting the immunogenic regions of HIV-1 envelope reduce mucosal infection and virus burden in humanized mice
Source: PLoS Pathog. 2022 Jan 5;18(1):e1010183. doi: 10.1371/journal.ppat.1010183 (PMC8765624; doi:10.1371/journal.ppat.1010183)
Supplement: S4 Fig — A) ELISA reactivity of 2219 WT vs Fc mutants was tested against recombinant gp120 JRFL. B) Neutralization of JRFL by 2219 WT vs Fc mutants was assessed with TZM.bl target cells after 24-hour mAb-virus incubation. CD4bs-specific bNAb NIH45-46 and irrelevant mAb 860 were included as controls. C) Concentration of 2219 WT vs Fc mutants in plasma of mice after passive infusion with each mAb (700 μg x 2 doses/animal, intraperitoneal, days 0 and 2). (PDF) [file ppat.1010183.s004.pdf]

Fig S4

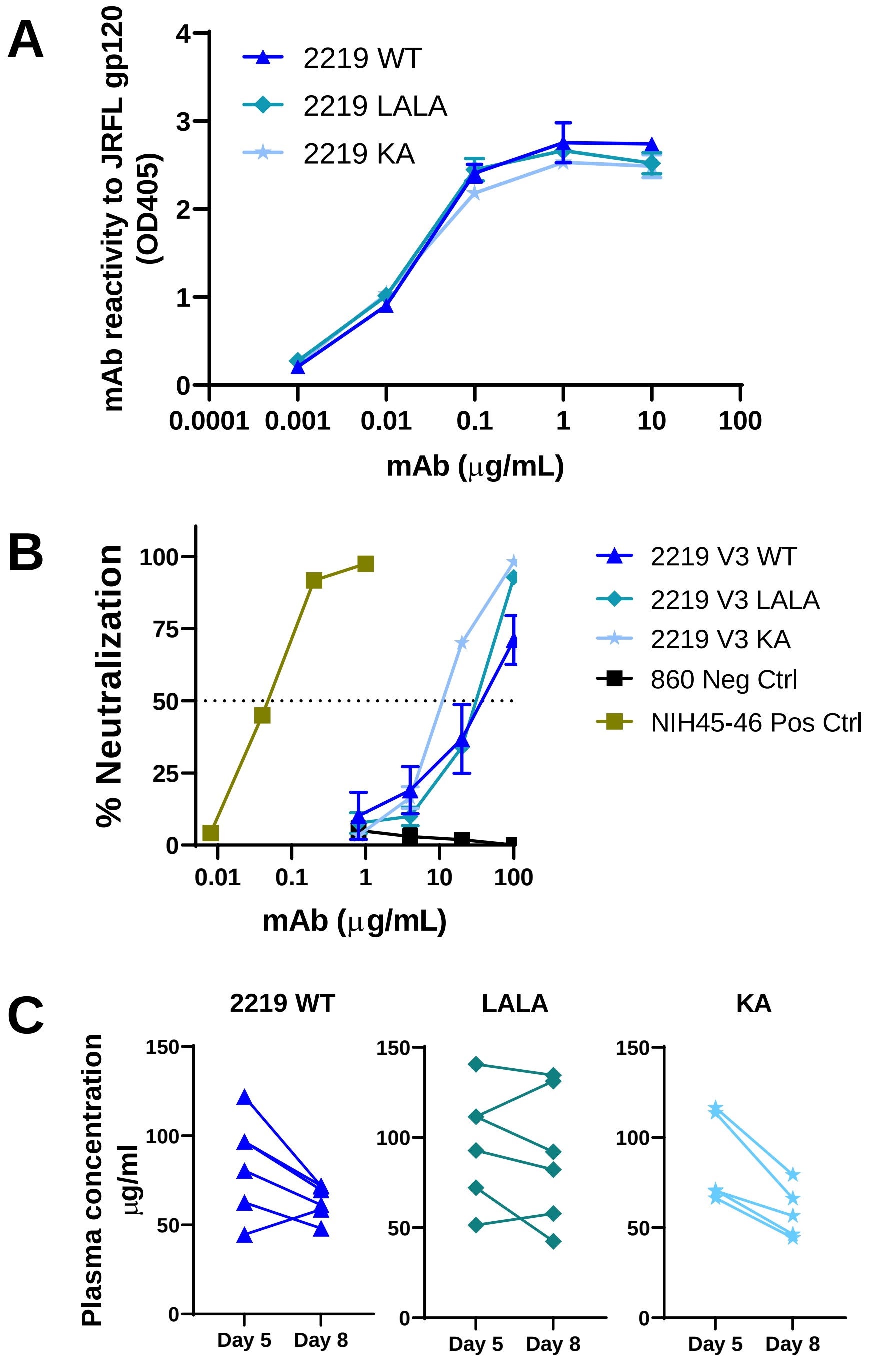

Fig S4: Fc mutations LALA and KA do not alter gp120 binding, neutralizing activity, or plasma concentration of V3 mAb 2219.

A) ELISA reactivity of 2219 WT vs Fc mutants was tested against recombinant gp120 JRFL.

B) Neutralization of JRFL by 2219 WT vs Fc mutants was assessed with TZM.bl target cells after 24-hour mAb-virus incubation. CD4bs-specific bNAb NIH45-46 and irrelevant mAb 860 were included as controls.

C) Concentration of 2219 WT vs Fc mutants in plasma of mice after passive infusion with each mAb (700 µg x 2 doses/animal, intraperitoneal, days 0 and 2).
