# Supplementary material for: Non-neutralizing antibodies targeting the immunogenic regions of HIV-1 envelope reduce mucosal infection and virus burden in humanized mice
Source: PLoS Pathog. 2022 Jan 5;18(1):e1010183. doi: 10.1371/journal.ppat.1010183 (PMC8765624; doi:10.1371/journal.ppat.1010183)
Supplement: S5 Fig — Spleen cells were subjected to intracellular staining with anti-p24 mAb KC57 and staining for markers of cell viability, human CD45 (huCD45), mouse CD45 (mCD45), CD4 T cells (CD3+CD8-), and monocytes (CD3-CD11c-CD14+). p24+ cells were detected in CD4 T cells or monocytes gated from viable human cells (hCD45+ and mCD45-). Dot plots from representative animals in the treated and mock groups are shown. (PDF) [file ppat.1010183.s005.pdf]

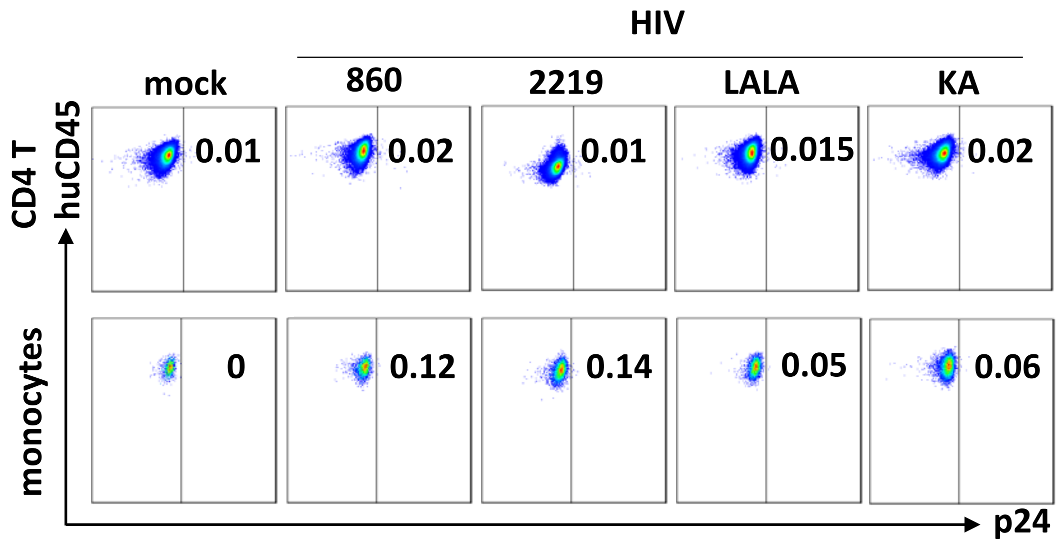

Fig S5. Flow cytometry for detection of p24+ cells in the spleen of humanized mice treated with 2219 WT or Fc mutants and challenged with JRFL IMC.
Spleen cells were subjected to intracellular staining with anti-p24 mAb KC57 and staining for markers of cell viability, human CD45 (huCD45), mouse CD45 (mCD45), CD4 T cells (CD3+CD8-), and monocytes (CD3-CD11c-CD14+). p24+ cells were detected in CD4 T cells or monocytes gated from viable human cells (hCD45+ and mCD45-). Dot plots from representative animals in the treated and mock groups are shown.
