# Supplementary material for: Non-neutralizing antibodies targeting the immunogenic regions of HIV-1 envelope reduce mucosal infection and virus burden in humanized mice
Source: PLoS Pathog. 2022 Jan 5;18(1):e1010183. doi: 10.1371/journal.ppat.1010183 (PMC8765624; doi:10.1371/journal.ppat.1010183)
Supplement: S1 Table — (PDF) [file ppat.1010183.s006.pdf]

Supplemental Table S1. Passive transfer experiments with V2i and V3 mAbs in human CD34+ HSC-engrafted mice.

Experiment I40	Group	Mouse ID	hCD45[1]	Plasma vRNA (copies/ml)				Spleen		Bone marrow		Lymph nodes	
				day 2	day 4	day 7	day 9	vRNA[2]	vDNA[3]	vRNA[2]	vDNA[3]	vRNA[2]	vDNA[3]
	No mAb+mock	2798	57.5	<10	<10	<10	<10	<0.001	<4	<0.001	<4	<0.001	<4
	Control mAb 860	2774	71.9	10707	15454	14588	16449	0.023	13492	124	83	1894496	274841
		2775	55.7	8668	2341494	51219	7386	0.033	91205	9	32	1963	179486
		2776	66.8	13812	8172	8086	5476	0.102	116781	17	63	319	3717818
		2777	53.3	33192	385702	3450	6250	60	106680	71	45	92	374221
		2799	32.8	40480	6833	51924	973599	217	178666	2022	191583	4084	1431583
	V2i mAb 2158	2770	60.6	10359	9274	160	5466	0.002	55	8	39	6	38
		2771	46.0	14100	1420	42999	25076	0.028	440	<0.001	<4	<0.001	1533577
		2772	78.8	5658	644	817	1687	<0.001	24	<0.001	<4	0.908	61
		2773	47.7	4842	539	3223	9887	0.043	190	<0.001	41	48	1648369
		2778	69.3	4371	2643	20741	6732	0.064	210	<0.001	7	103	1659049
	V3 mAb 2219	2805	63.1	2391	<10	<10	<10	0.002	<4	<0.001	<4	<0.001	<4
		2806	59.7	2023	3208	541	<10	<0.001	<4	<0.001	8	6	29059
		2807	71.1	8925	<10	<10	<10	<0.001	<4	<0.001	<4	<0.001	<4
		2808	35.5	5705	3630	1487	12071	0.004	<4	1	<4	0.511	4185
		2809	56.5	1404	<10	<10	<10	0.070	<4	<0.001	<4	0.309	<4

Experiment I63	Group	Mouse ID	hCD45[1]	Plasma vRNA (copies/ml)			Spleen	
				day 5	day 8	day 17	vRNA[2]	vDNA[3]
	No mAb+HIV	4032	9.2	12047	28137	1546	<0.001	<4
		4040	15.1	27333	54311	101	523	<4
	Control mAb 860	4030	51.8	11008	7186	41688	13	<4
		4031	39.0	<10	387831	752	2	<4
		4033	62.8	7654	7018	<10	1	5
		4034	54.9	<10	122668	3327	<0.001	<4
		4035	27.2	<10	11095	<10	13	<4
		4036	16.9	8414	n/a	3803	55	<4
	V3 mAb 2219 WT	4037	39.5	24395	67568	267	<0.001	5
		4038	38.3	29023	11522	<10	<0.001	<4
		4039	37.6	9372	16922	697	<0.001	54
		4041	48.0	45898	10778	14217	10	<4
		4042	70.8	<10	13563	6801	51	127
		4043	32.6	10157	20875	2730	136	159
	V3 mAb 2219 LALA	4044	59.3	22635	56505	3757	<0.001	39
		4045	43.4	<10	148731	<10	<0.001	<4
		4046	45.5	16360	5379	1513	1.7	<4
		4047	36.6	18963	11727	<10	6.2	<4
		4048	45.4	19934	43402	3085	<0.001	<4
		4049	26.4	22486	32636	<10	<0.001	<4
	V3 mAb 2219 KA	4050	50.4	<10	8763	1035	2	<4
		4051	30.4	18922	61995	8150	<0.001	<4
		4052	28.6	25038	20555	1642	63	<4
		4053	44.4	<10	179196	<10	0.264	<4
		4054	37.6	13086	39824	<10	41	<4
		4055	30.1	32584	354017	<10	50	<4

Experiment I70	Group	Mouse ID	hCD45[1]	Plasma vRNA (copies/ml)				Spleen		Spleen[4]	
				day 2	day 6	day 9	day 14	vRNA[2]	vDNA[3]	$p24^+$CD4 T	$p24^+$ mono
	mock	4433	43.4	<10	<10	<10	<10	<0.001	<7	0	0
		4436	54.3	<10	<10	<10	<10	<0.001	<7	0	0
	Control mAb 860	4419	44.9	9331	12203	7098	2595	290	639.9	0.016	0
		4420	36.6	21233	6440	6229	1419	2354	144.7	0.016	0.120
		4421	52.7	1339	9813	5250	11348	3601	91.9	0.025	0.017
		4423	32.3	2876	6492	11932	1093	523	95.6	0.014	0.200
		4435	47.1	14502	13958	7608	2380	246	31.2	0.020	0.036
	V3 mAb 2219 WT	4424	43.1	578	6129	6015	0	10	30.4	0.009	0.023
		4428	33.8	<10	11193	6420	1610	2	7.9	0.013	0.410
		4429	32.5	6177	5810	8014	0	8	57.8	0.009	0.140
		4431	31.3	1514	12017	6068	1025	236	130	0.006	0.070
		4432	39.4	9393	9611	2084	638	267	119.8	0.007	0.320
	V3 mAb 2219 LALA	4425	39.0	629	10011	4299	0	49	43.3	0.012	0.029
		4426	38.3	<10	10531	4112	0	10	45.7	0.014	0.047
		4427	44.9	23892	7946	4704	746	20	34.4	0.015	0.067
		4440	16.4	2496	11643	6454	1852	8	48.7	0.015	0.094
		4442	40.5	2022	5907	7883	0	74	47.7	0.012	0.025
	V3 mAb 2219 KA	4437	13.6	758	11326	6841	0	8	67.9	0.017	0
		4443	63.3	12985	7774	12287	2267	232	46.5	0.021	0.083
		4445	49.2	3511	12397	2370	1057	59	288.4	0.022	0.064
		4446	35.3	7740	8676	4941	776	10	29.3	0.019	0.140
		4447	47.1	7681	8477	7582	3521	97	13.6	0.021	0.095

[1] % human CD45+ cells in the blood prior to treatment

[2] Relative fold

[3] copies/10^6 human CD45+ cells at the end of experiment

[4] % $p24^+$ cells in human CD4 T cell or monocyte populations
