# Supplementary material for: Non-neutralizing antibodies targeting the immunogenic regions of HIV-1 envelope reduce mucosal infection and virus burden in humanized mice
Source: PLoS Pathog. 2022 Jan 5;18(1):e1010183. doi: 10.1371/journal.ppat.1010183 (PMC8765624; doi:10.1371/journal.ppat.1010183)
Supplement: Attachment — Submitted filename: Response to Reviews - 11 22 2021.pdf [file ppat.1010183.s007.pdf]

Responses to Reviews 1 and 2

Summary
Reviewer #1:
The manuscript by Hioe et al tests an important hypothesis about the role of non/poorly neutralizing antibodies in mediating clinically relevant antiviral activities of HIV-specific antibodies in a humanized mouse model. The work is generally well and thoroughly done. The results are impactful and timely.

Reviewer #2:
This manuscript analyzes the protective effect of two non-neutralizing mAbs directed to highly immunogenic epitopes V2i and V3 regions of HIV. The effect of non-neutralizing Fc-mediated inhibitory functions in HIV protection is highly suspected but not firmly demonstrated. In fact, in vivo animal models have many limitations, hampering the study of the role of FcR-functions in vivo. The humanized mouse model used in this study has the advantage to display humanized immune cells with the exact human FcR expression.
In this manuscript, the FcR function in HIV protection was extensively introduced and nicely discussed, giving a complete and comprehensive overview of the current knowledge in the field. The results of this study support previous studies, again suggesting the involvement of Fc-mediated antibody functions in in vivo decreased virus load. In this study however, authors show that Abs directed against V3, a highly immunogenic epitope, was found to have partial protective effect in vivo. Moreover, they could associate this decreased viral load the Fc domain of Ab and with in vitro ADCP function. This study is therefore relevant as it enlarges the epitopes and Fc-mediated function of Abs potentially involved in in vivo decreased viral load.

> **Response**: We thank the reviewers for these constructive and thorough reviews. We are pleased that the data presented in our paper are found to be relevant, impactful, and timely.

Minor Issues: Editorial and Data Presentation Modifications
Reviewer #1:
1) IgG3 correlate in RV144, and association of IgG3 with elevated ADCP, may be worth specific mention in the introduction. Also may be worth mentioning HVTN 505 case-control correlates results (for example, elevated ADCP).
Response: We have added statements about IgG3 and ADCP as correlates in RV144 vs HVTN505 plus the pertinent references in the Introduction (page 6).

2) WNV and influence non-nAb mentions are out of place in introduction. Also unclear why those and not other viruses are mentioned.
Response: The reviewer is correct that the sentence is out of place and irrelevant. We have removed it from the Introduction (page 6).

3) Consider acknowledging the diversity of ADCC assays, which often show different activities for mAbs. These Abs may exhibit different relative activities in other ADCC assays.

Response: We have added statements to acknowledge this possibility in the Discussion (page 22).

4) Based on how the affinity measurements were made, they likely reflect avidity measurements (bivalent).
Response: We have clarified the affinity measurement in the Materials and Methods section (page 30) and in the **Figure 2C** legend. Each mAb was immobilized on Anti-hIgG Fc Capture (AHC) biosensors and reacted with monomeric JRFL gp120 protein in solution at the designated concentrations. This experimental condition measured the affinity of each Fab fragment for gp120 in a 1:1 stoichiometry.

5) The authors should address the disconnect between NHP and humanized mouse models. For example, PGT121 dependence on effector function differs between models. This should be discussed as the finding of KA importance may be unique to mouse. What do the authors suppose explains the non-reduced activity of LALA, which also reduces complement activation?
Response: We have added statements in the Discussion (page 21) to address the issues about disparate results from bNAb passive administration in NHP and mouse models.

In regard to the activity of LALA, we have now clarified in the Discussion (page 21) and in the Results (page 19) that LALA mutations which decreased ADCP more than complement activation did not have as much effect in vivo as the KA mutation which abrogated only complement binding. The greater effects of KA vs LALA observed in vivo corresponded best with a greater reduction of complement binding by KA vs LALA mutations.

6) Comment on biological relevance of readouts that were and were not statistically impacted by mAb administration.
Response: We have revised the Results section describing **Figure 6** (pages 18-19) to indicate the readouts which were and were not significantly impacted by 2219 WT vs KA or LALA variants. We also revised and added statements in the Discussion (page 21) to comment on the relevance of partial effects seen with KA and LALA mutations.

7) The caveats of assessing complement activities in vitro that are relevant in vivo should be clearly acknowledged. For example, the ability of Ab to link gp120 on an ELISA plate to C1q binding does not mean that that same antibody will have any complement activity in other assays (or in vivo).
Response: We have now included these caveats in the Discussion (page 21).

8) The authors should be careful to draw attention to the divergence in LALA and KA phenotypes relative to their nominal phenotypes. Ie: LALA was only an intermediate knockout of C1q binding in one of the two complement assays used.
Response: We revised sentences throughout the manuscripts to clearly state that LALA diminished or reduced ADCP and complement deposition (e.g. page 17). We also clarified that

LALA decreased ADCP more than complement binding, while KA abrogates complement binding without affecting ADCP.

9) Can the authors comment on the reproducibility of ADCP data in fig 5A? The KA titration curve appears to have no error bars and strong hook effect. Understanding the reproducibility of that dose response profile may contribute to its interpretation.
Response: Thank you for pointing the missing error bars for the KA titration curve in **Figure 5A**. We have now included error bars for each of the titration curves in this graph. The ADCP data in **Figure 5A** were seen reproducibly in repeat independent experiments. Data from one of these experiments are presented. The prozone or hook effect is often seen in our and other ADCP assays and is displayed by mAbs and polyclonal samples with high ADCP potencies (e.g. Klingler et al. https://www.medrxiv.org/content/10.1101/2021.05.11.21256972v1.full; Butler et al. https://doi.org/10.3389/fimmu.2019.01851; Herter et al https://pubmed.ncbi.nlm.nih.gov/23873847/).

10) Authors should comment on statistical and biological significance, especially in light of many readouts having large spread, and some experiments having relatively few animals (ie: fig 6D) (suggesting limited resolution to meet arbitrary significance thresholds).
Response: We have added statements in the Results sections to point out the lack of statistical significance in light of the small samples size in **Figure 6D** (page 19) and the high variability of virus burden among individual animals (**Figure 1** page 11, **Figure 6** page 18).

11) Why is there no experimental variance in the mAb 860 virus capture? (Fig 3B)
Response: Data from 2-3 repeat experiments were compiled for this graph, and virus capture was calculated relative to control mAb 860 included in each experiment (set to 1). This information is included in the **Figure 3B** legend. In addition to 860, purified human HIV-seronegative IgG sample (IgG-bkg) were also tested as negative control and showed experimental variance. Statistical analysis was done by comparing V2i and V3 mAbs with IgG-bkg.

Reviewer #2

1) A few question nonetheless rises. Are the two mAbs used in this study produced by reconstitution of the Fc heavy chain domains therefore leading to the same Fc reconstituted domain for the two Abs, or have these mAbs the heavy chain as isolated from the patient? In the second case, the capacity of the Fc domain to bind to FcR may also varied considerably for these two Abs, therefore affecting FcR mediated functions. Consequently, not only the Fab domain recognizing the virus but also the Fc domain binding to FcR may influence FcR functions. This point need to be discuss especially for ADCC results.
Response: Both V2i mAb 2158 and V3 mAb 2219 were produced as recombinant IgG1 in transfected 293F cells. This information is provided in the Materials and Methods section (page 28). The complete plasmid sequencing has been performed to verify that indeed their Fc heavy chain domains are identical.

Additional comments:
2) The RNA as AUC, vRNA in spleen or cell-associated RNA measured following 2219 treatment in figure 1C, D, E suggest two populations of mice: responders versus non responders. This heterogeneity may also be the results of difference of virus replication in the 3 independent experiments performed. Indeed, according to supplemental table 1, virus replication was lower in control group of experiment 63. Colors codes of the dots according to the experiment figure 1C, D, E may help to decipher this point.
Response: We appreciate this suggestion. We prepared **Figures 1C, D, E** in which individual animals from each of the 3 experiments were marked: completely filled symbols for Experiment I40, half-filled symbols for Experiment I63, and open symbols for Experiment I70 (**see Figure 1 below**). Only vDNA levels in the 860 group in **Figure 1E** clustered according to the experiments. In the rest of the 2219 and 860 data sets in **Figures 1C, D, and E**, the data points from different experiments are dispersed with no apparent clustering. Therefore, we opted to keep the **original Figures 1C, D, and E**.

3) To assess ADCC, authors performed 3 different in vitro assay. In order to better understand the outcome of each assay and facilitate understanding, the principle and limitations of each ADCC assay may be summarized in a table.
Response: We have now included a table (**Supplemental Figure S3 E**) that summarizes the experimental details for FcɣR signaling and ADCC assays performed in this study.

Figure 1

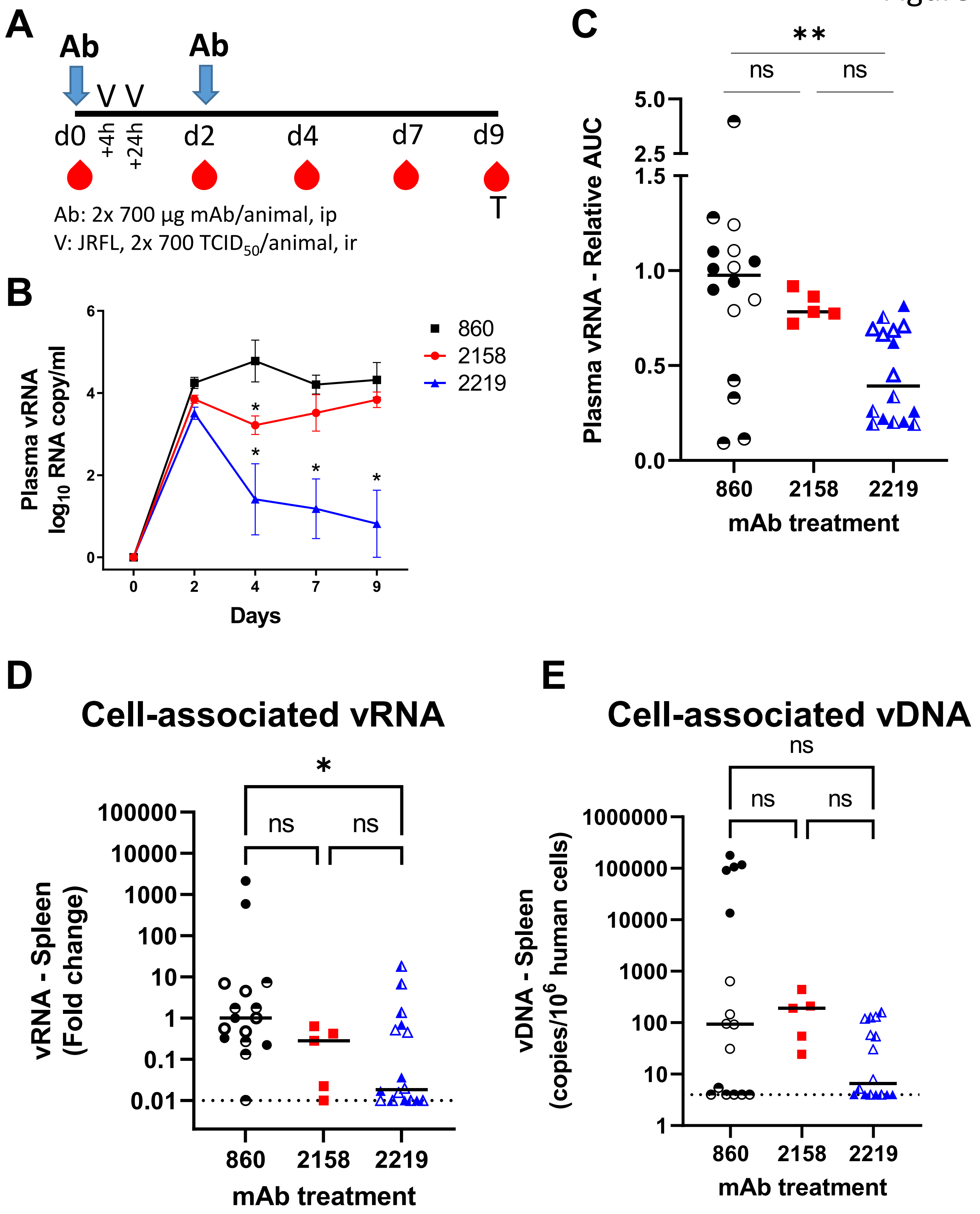

Completely filled symbols: Experiment I40
Half-filled symbols: Experiment I63
Open symbols: Experiment I70

Figure 1: The capacity of V2i mAb 2158 and V3 mAb 2219 to reduce virus infection in CD34+ HSC-engrafted humanized mice upon rectal challenge with HIV-1 JRFL IMC.

A) Schematic of experimental protocol showing that mice received intraperitoneal administration of mAb at day 0 and were challenged rectally with HIV at 4 hours and 24 hours. Animals received the second dose of mAb at day 2. Virus dose (2x700 TCID50 per animal) was pre-determined to yield infection in all control mice. Blood and tissue samples were collected at the designated days for measurement of vRNA and vDNA in individual animals.

B) Mean plasma vRNA loads from day 0 to day 9 in each group of animals that received control mAb 860, V2i mAb 2158, or V3 mAb 2219. Data from one of three experiments are shown.

C) Plasma vRNA loads of individual mice in each of the three groups. Area under the curve (AUC) of vRNA over time was calculated. Data are presented as relative AUC over mean AUC of the control 860 group included in each experiment. Three experiments were performed with cohorts of mice engrafted with different HSC donors. N=16, 5, and 16 for mice treated with 860, 2158, and 2219, respectively.

D) Relative levels of cell-associated vRNA detected in the spleen collected at the end of experiment from individual mice in the three groups. Data from three experiments are compiled and presented as fold changes over median vRNA of the control 860 group in each experiment.

E) Cell-associated vDNA levels in the spleen of mice from the three groups. Data are shown as vDNA copies per 10^6 human CD45+ cells.

Statistical analysis was done with Kruskal Wallis one-way ANOVA test with Dunn’s multiple comparison. **, $p<0.01$; ns, not significant ($p>0.05$). Horizontal bars: median. Dotted lines: detection limit.
